# Reverse engineering of ceramic anthropomorphic figurines from the Tumaco archaeological tradition in southwest Colombia

**DOI:** 10.1371/journal.pone.0250230

**Published:** 2021-05-05

**Authors:** Nohora Alba Bustamante, Jairo Arturo Escobar, Marcos Martinón-Torres

**Affiliations:** 1 Department of Mechanical Engineering, Universidad de los Andes, Bogotá, Colombia; 2 Department of Archeology, University of Cambridge, Cambridge, United Kingdom; University at Buffalo - The State University of New York, UNITED STATES

## Abstract

Traditional studies of archaeological ceramics in Colombia have been largely based on visual and stylistic analyses. Here we introduce frameworks and concepts of reverse engineering as a complementary strategy to develop hypotheses about ceramic manufacture, as a first step to the address possible cross-craft relationships and broader sociocultural parameters affecting technical traditions. Our case study is focused on ceramic figurines recovered from two archaeological sites in southwest Colombia (Inguapí and La Cocotera), both dated to the period of greatest cultural and technological development of the Tumaco tradition (350 BC–AD 350). The results of the analyses including microscopy, XRF, SEM-EDS and XRD revealed two manufacturing pathways within the broader tradition, developed locally and adapted to the natural resources available to each site. These are shown through chemical and mineralogical differences in the raw materials, as well as differences in their preparation and shaping, molding, and modeling processes as observed at the microstructural level. Estimated firing temperatures are under 600°C for La Cocotera, and under 800°C for those of Inguapí, with an inhomogeneous, oxidizing atmosphere probably related to firing in a pit. The superficial characterization shows that all the figurines were painted, with those from Inguapí externally smoothed and polished, and those from La Cocotera covered with a slip. Notwithstanding differences between sites, the ceramic figurines illustrate a particular technical style that undoubtedly conveyed a shared ideological message of cultural affiliation. These results contribute in an innovative way to archaeological ceramic studies in Colombia from a different perspective that is complementary to the more common typological studies.

## Introduction

The Tumaco archaeological tradition is part of a homogeneous cultural complex known in Colombia as Tumaco and in Ecuador as La Tolita. This tradition reveals valuable information on the human use of tropical jungle environments since it geographically extended for about 500 kilometers along the Pacific coast, from Buenaventura in Colombia to Atacames in Ecuador [1]. The evidence obtained by the archaeological studies conducted on the Colombian side established a regional sequence of four phases extending from 500 BC to 1500 AD. The first phase, ranging from 500 BC to 350 BC, was referred to as Inguapí I by Patiño (2003), corresponded to the earliest occupation, when people, thought to be originally from Ecuador [2–6], settled on mangrove and, interfluvial areas. Few solid and hollow ceramic anthropomorphic figurines were found, mostly in the deepest strata. These were somewhat stylized and with few ornamentations, mostly modeled and molded in a fine paste, and depicted mostly female characters dressed in simple way [7–9].

A new ceramic style referred to as Inguapi II by Patiño (2003) appeared during Tumaco´s second phase. Although excavations do not show abrupt stratigraphic interruptions, key changes are identified, namely an increase in the number and size of settlements with clear evidence of hierarchization, expansion of these settlements in all the physiographic zones of the region (mangroves, interfluvial, alluvial and flood plains), higher complexity in the pottery industry manifested in the variety of techniques, forms, decorations and finishing, as well as mass production of ceramic figurines, development of platinum and gold metallurgy, intensification of exchange in prestigious goods, construction of artificial mounds or *tolas* as ceremonial centers, and elevated cultivation fields with channels allowing the development of intensive agriculture [7, 10]. This phase lasted from 350 BC to 350 AD and, it is considered a climax of social and technological complexity of this tradition [5–7, 10, 11].

A considerable number of figurine fragments recovered during this phase, found predominately in domestic and residential contexts, was also found in the context of middens, funerary, and ceremonial contexts [7, 10, 12–15]. Descriptions obtained from excavation reports are brief, general and without exception mention that figurines belong to a diagnostic style known in Colombia under the name of "Figurillas Tumaco" and in Ecuador as “Figurillas La Tolita” [5, 7, 9, 10, 12, 13, 15, 16]. The common characteristics described by different researchers include: a low proportion of figurines findings (between 0.3% and 2%, when compared with the total quantity of ceramics recovered [7, 10, 13, 15]); they mainly depict naturalistic human figures in standing position or sitting on a stool; and in a lesser number zoomorphic figures and hybrid or fantastic beings [7]. All have ornaments, some represented in ceramic, others suggested through holes in the ears or nose that probably held objects worked in other materials such as wood, shell or metal. These ornaments are also morphologically similar to the larger metal counterparts. Some figurines show simple ornaments such as ear-plugs, nose rings, necklaces, hood-style headdresses and simple tunics while others were are highly decorated with personal ornaments and attire of a greater complexity, with areas or stripes painted in red. The size of the pieces is variable, with faces ranging between 4 and 14 cm, indicating figurines of different sizes [13]. Some figurines were additionally designed to be used as musical instruments, some representing musicians playing *ocarinas*, whistles and drums [7].

The previously mentioned characteristics have been complemented with detailed descriptions of nearly 4500 figurines, focusing on stylistic and iconographic examination [9, 17–20]. These studies confirm the type of representation previously described but add more details to each type: anthropomorphic characters represented include men, women, couples and families, heads and masks; zoomorphic characters such as toads, armadillos and butterflies were less naturalistically represented; animals like owls, monkeys, opossums and especially felines and other predators with body ornaments such as sharks and caimans were highly represented. Domestic fauna commonly found in Tumaco—La Tolita refuse middens was not part of the iconographic universe [8]. The type corresponding to hybrid beings, commonly appears in highly decorated with hanging tongues and headdresses exclusive to this type of representation. They are different from the ones used by the anthropomorphic figures and share attributes of a fantastic nature not readily attributable to any animal species.

Representations of females are commonly naked and it is believed that they are indicative of motherhood, due to the presence of pregnant figures and figurines where a figure interpreted as female is observed holding a child [21]. Female representations are more uniform in dress and ornaments, which are simple in nature, possibly representing everyday use. They were adorned with simple hooded headdresses and show a hieratic posture. Women figurines rarely appear portraying any kind of activity [22]. They are small in stature, making them more accessible as portable objects to be used as votive cult figures. They are presumed to have been used in domestic contexts to celebrate one or several female deities that existed in the earlier chronological period [8, 23].

Male figurines were recovered in greater numbers and vary more broadly in their iconography, size and, attire when compared to female figurines [9]. They are frequently depicted in ceremonial contexts and are thought to represent distinctive statuses, ranks or offices. They are commonly found seated on stools; adorned in helmeted headdresses; wide necklaces with rectangular hanging plates; bracelets, anklets, and fight-fitting belts under the knees. They are also clothed with loincloths, ponchos and feathered costumes that are presumably may symbolizing the action of dance. Male figurines are also adorned with feline masks or zoomorphic headpieces depicting felines, birds, sharks, owls, or fantastical beings. Many are noted for representing specific trades, such as canoeists, basket makers, musicians, dancers, and shamans [8, 19, 21].

Researchers have drawn hypotheses concerning the meaning and use of the figurines through tools such as semiotics, ethnography, biological analogy, and synchronic and diachronic comparative analyses. However, they are limited by the lack of written sources, inconsistencies in both the time frame and the cultural codes, and particularly the absence of archeological contexts for most of the figurines related to this tradition [9, 13, 15, 17–21]. Despite these limitations, the evidence suggest that the figurines are complex and organized symbolic representations, which show some consistency in their morphological features [9, 21, 22]. This reflects their political and religious nature [13, 14]. Furthermore, their change throughout time depends on the gender of the represented character: while feminine figurines remain almost unchanged, masculine representations increase in complexity [9].

The continuity of the female representations has been used as a basis to suggest that the social role of women remained broadly similar throughout the Late Formative and this phase [9, 13, 25]. For instance, these artifacts acted as a metaphor for creation and regeneration, which relate to the reproductive power of the female body and are directly associated with agricultural cycles [17, 21, 24]. This interpretation is consistent with the intensification of the agricultural production that was observed during these periods [13]. Moreover, the simplicity of female attires has been taken to suggest that women were excluded from active social rituals such as dancing or shamanic ceremonies [9, 16]. In contrast, these figurines would indicate that women served as assistants during either healing, protection or domestic rituals, or worship of health, family, and maternity related deities [19, 24].

In contrast with female representations, the changes in quantity, size and representation observed in male figurines are interpreted as indicative of their social and political roles [9]. First, an increase in the number of masculine figurines may be related to a higher dominance of this gender in the Tumaco-La Tolita society [9]. Second, the enlargement of male figurines suggests a ceremonial function as part of collective cult [9, 19]. Third, male representations varied in posture, attire, and activities. For instance, with reference to ethnographic examples, hybrid beings with complex headdresses and hanging tongues are thought to represent common ancestors and descent. Therefore, the new roles represented by men during this phase indicate an increased social complexity in a community that gradually became more hierarchical, where the religious activities and kingship of an individual were the main determining factors for achieving a high position in this society [9,19].

Few figurines pertain to the end of Inguapi II were found and those that were recovered archaeologically are crude, handmade, and simply adorned figures. The simplicity of the figures has been taken as an indication of the transition to the third phase, which spans from around 350 AD to 600 AD [7]. Archaeological evidence suggests a 200-year chronological gap, with the fourth phase extending from ca. 800 AD to 1500 AD. Anthropomorphic figurines from this time are simple and plain with schematic representation of the human form [7, 25].

Material culture of the Tumaco-La Tolita tradition is widely recognised in archaeological literature and pre-Hispanic art in America [13, 26, 27], and numerous ceramic and metal objects, including in platinum and gold, are on display in museums around the world [28]. Ceramic figurines are notable for the display of manufacturing techniques such as molding and modeling, or the combination of both. Additionally, platinum and gold objects reveal unusual artistic skill, as well as exceptional knowledge in handling these materials and the implementation of innovative technologies to process them. It is worth noting that platinum processing was not possible in Europe until the 18th century [29], while the metallurgists of Tumaco-La Tolita tradition were able to work this metal since 350 BC [30].

In both materials, whether metal or ceramic, a particular "style" is recognized, as a hallmark of this tradition that undoubtedly combined sociocultural, symbolic, political, religious, environmental and economic factors with successful technological processes, and which involved technical decisions that are culturally embedded [31–33]. An increase in funeral offerings and demand for paraphernalia, led to the elaboration of personal ornaments with new materials available in the region, such as gold and platinum, possibly as an indicator of status and included procurement of clay for the making of the figurines, polished stones, obsidian, rock crystal and coca leaves.

This evidence could be the consequence of the institutionalization of the shamanic practice [34] and the intensification and the development of ritual practices. These practices were in ceremonial centres for larger and more complex groups. The figurines were larger scale, less appropriate as shamanic assistance but more suitable as icons for new ritual practices [21]. The above suggests socio-political and ideological changes, in communities with political leadership by kinship, the need for development and technological specialization to satisfy and support needs of its leaders [35].

In particular, Tumaco ceramic figurines illustrate a culturally cohesive tradition through a characteristic “style” in which the figurines show a naturalistic expression and anatomy, and motifs that are repeated with the same characteristics, produced by different artists who apparently do not appear to have had much creative freedom [9]. Iconography cannot be considered arbitrary, but rather the product of a culturally determined requirements; as such they were an active means of a communication, used to transmit ideological messages to a large number of people across extensive geographic areas [19, 36].

These messages can be categorized according to the iconographic repertoire. The first category, which corresponds to most of the ceramic materials, depicts domestic scenes and activities that reflect the life cycle [19, 20, 37]. A second category comprises figurines related to ritual activities, with exaggerated ornaments, postures and typical elements of authority and power. The third category refers to sexual distinctions and the role that men and women played as social actors [2, 4]. Consequently, the ceramic iconography conveys information about the social structure and formed part of the religious political power system of this tradition [9] Identifying figurines´ context is paramount in determining how these materials were used and how they were produced.

Trying to solve any question around these figurines must consider the context in which they were recovered. In this case, archaeological evidence places the Tumaco-La Tolita figurines mainly in middens, and to a lesser extent, in funerary contexts [7, 19]. The presence of such figurines in middens suggests their use in residential or domestic contexts, probably as votive figures used for the cult of motherhood, health or agriculture. Likewise, their occurrence in funerary contexts is an indicator of ceremonial activities at the community level [8, 9, 22].

The style evident in the figurines is a product of cultural action, but the materialization of those actions involved a series of activities and technical choices that reflect the potters’ knowledge, ability, experience and environment. Considering the mass production of similar-looking ceramic figurines over a ~ 500 km stretch of geography and for over 700 years, it is appropriate to ask the extent to which technical choices show technological variability or homogeneity. This question has implications for our understanding of the organization of production: for example, high standardization may be reflective of concentration of production and distribution centers, whereas higher variability would be expressive of local or regional manifestations of a shared ideological message.

Against this background, this research puts forward the use reverse engineering to formulate hypotheses of ceramic figurine manufacturing in the Tumaco tradition, in order to offer an evidence-based reconstruction of ceramic production technology and technological choices. We will focus specifically on two sets of ceramic figurines of similar styles but recovered at two different sites. This information will aid our comparative understanding of the transformation of raw materials into cultural objects in their sociocultural, environmental and technological context. At the same time, we propose a framework that should be applicable to other case studies, while contributing to the much-needed development of science-based studies of archaeological ceramics in Colombia.

## Methodology, samples and experimental techniques

The methodological framework used for the archaeometric analysis of the fragments is that broadly known as "reverse engineering". This is a recognized methodology used in different fields of knowledge such as forensic engineering, failure analysis, medicine, or art, and recently introduced in biomimetics and heritage studies. This methodology allows researchers to identify and characterize evidence in artefacts at different scales, compositional, mineralogical, textural, microstructural, using different materials science and engineering tools. The integration of these structural characteristics serves as the basis for the formulation of manufacturing hypotheses [38] and in turn allows to interpret technical choices used in the production sequence of these objects [31].

**Fig 1:**
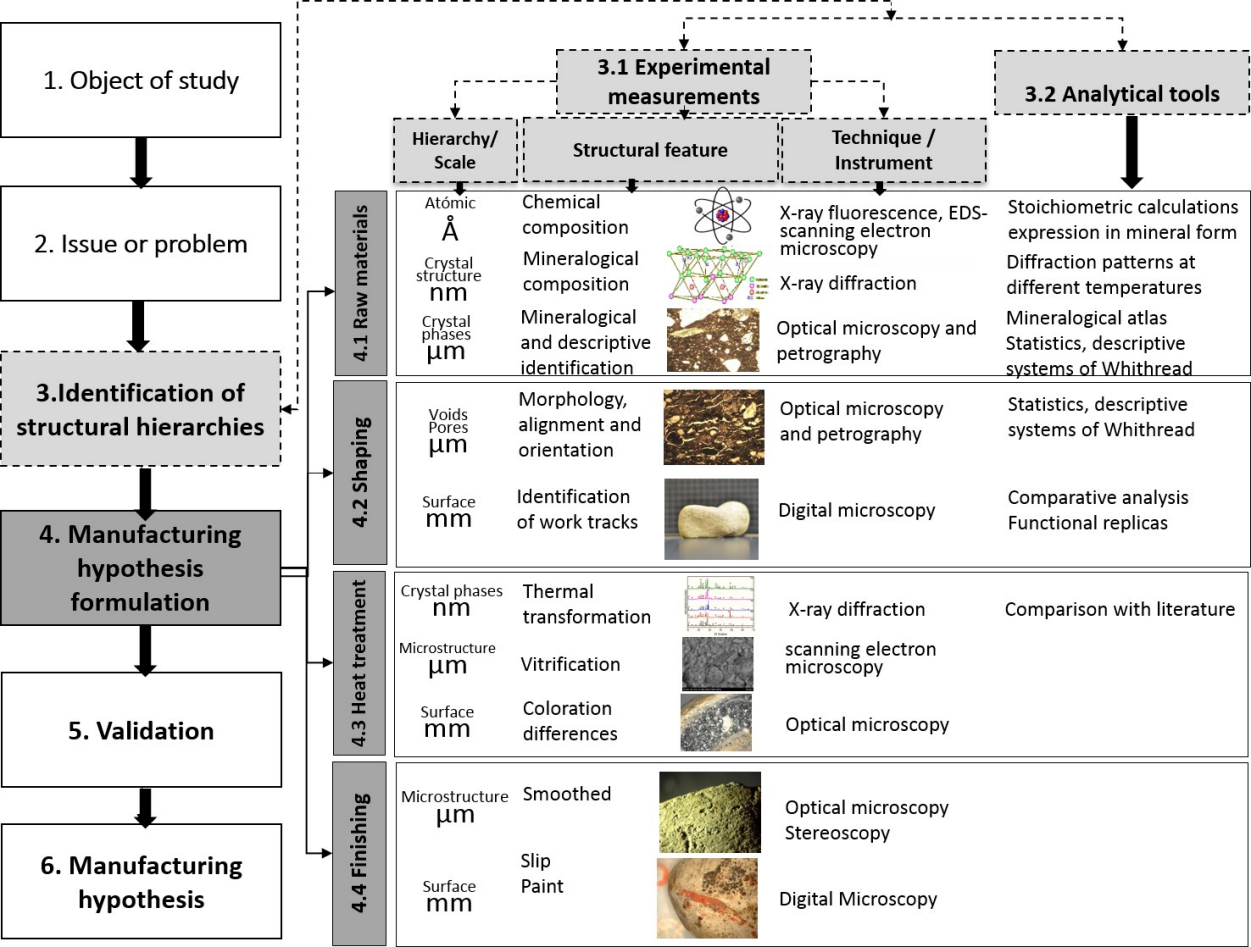
Methodological stages implemented to postulate the manufacturing hypotheses of the studied potsherds using a reverse engineering framework.

For its implementation, the six methodological stages shown in Fig 1 were followed. The starting point was the selection of the study sample accompanied in tandem with the research question, followed by the identification of the structural hierarchies and the formulation of hypotheses. These stages are followed by validation, which involves an iterative and experimental process in which comparative data is provided to finally refine the proposed manufacturing hypotheses.

In *Stage 1* (Fig 1) the study sample was selected, for which the following criteria were considered: First, the selected fragments were materials collected by two research projects, duly contextualized in the geographical of the Tumaco tradition, hence allowing a comparison of products, manufacture stages and thus technological choices. Second, that selected samples had similarities in terms of representation, chronology, ecosystem and context: regarding representation, all are anthropomorphic figurines or sherds; chronologically, they all belonged to the second phase (350 BC–AD 350), where we document the greatest cultural and technological development, as confirmed by radiocarbon dating of associated samples and the presence of platinum-gold metal pieces associated with the ceramic figurine fragments; environmentally, all the samples were recovered from the same physiographic zone (mangrove, interfluvial, floodplain or fluvial plain) and the same contexts: middens, habitation sites, ceremonial centers, and/or funerary contexts. Third, the selected ceramic material must have the necessary permits to take samples for analysis.

**Fig 2:**
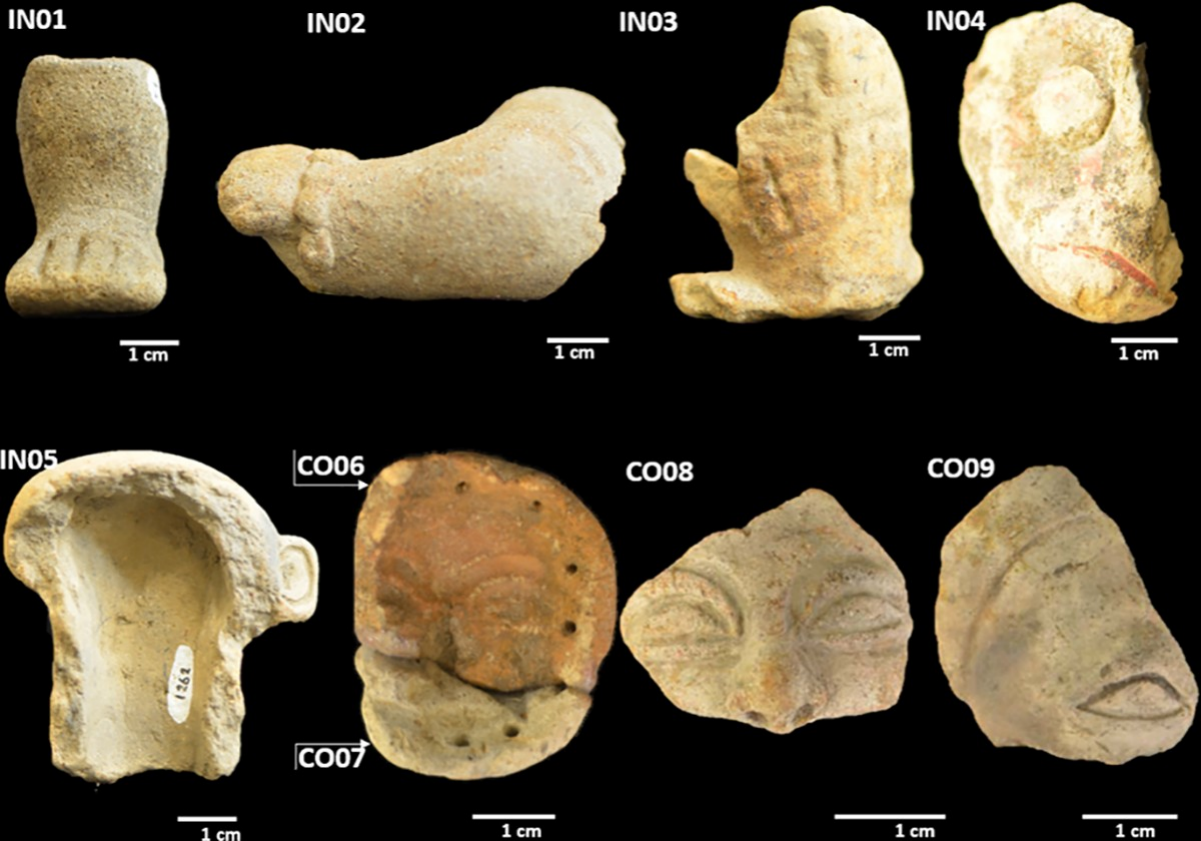
Figurine fragments selected for study. From left to right assigned identification/original identification IN01/FR-A0034 T1 Z16 5; IN02/FR-A0034 T1 Z16 16; IN03/FR-A0034 T1 Z16 14; IN04/FR-A0034 1261; IN05/FR-A0034 1262; CO06/R SUP 87 (dark area); CO07/R SUP 87 (bright area); CO08/Cocotera Tr4 20–30 cm and CO09/Coc. Sub. Tr4 50–60 cm.

The ceramic figurine fragments that met the selection criteria are shown in (Fig 2). The identification of such fragments and their main characteristics are summarized in Table 1. To each of the specimens, two identification numbers are shown: the one used in this work and the original designation from the studies that report the archeological findings of these fragments. In this paper, only the first ID number will be used.

**Table 1:** Identification and general characteristics of the analyzed fragments.

ID number	Characteristics
**IN01** FR-A0034 T1 Z16 5	• Fragment collected by Bouchard’s project, found in a midden, mound 5, trench 1, grid Z16, #5
	• Recovered from the mangrove physiographic zone.
	• Leg fragment of a ceramic anthropomorphic figure.
	• Height: 4.9 cm, Width: 4.9 cm, Length: 2.8 cm and walls between 8 mm y 9 mm.
	• The outer wall has a light cream colour and dark interior.
	• The surface is smooth and eroded. The piece was painted
	• Associated with the Inguapí ceramic complex and red paint decoration.
	• In site was found 3 gold threads and 38 foils with thicknesses between 0.05 mm and 0.2 mm of gold, silver and, copper alloy with platinum.
**IN02** FR-A0034 T1 Z16 16	• Fragment collected by Bouchard’s project, found in a midden, mound 5, trench 1, grid Z16, #16.
	• Recovered from the mangrove physiographic zone.
	• Arm fragment of a ceramic anthropomorphic figure.
	• Height: 4.3 cm, Width: 7.1 cm, Length: 2.8 cm and walls between 8 mm y 9 mm.
	• The outer wall is light cream colour and dark interior. The surface was smooth and painted.
	• Associated with the Inguapí ceramic complex and red paint decoration.
	• In site was found 3 gold threads and 38 foils with thicknesses between 0.05 mm and 0.2 mm of gold, silver and, copper alloy with platinum.
**IN03** FR-A0034 T1 Z16 14	• Fragment collected by Bouchard’s project, found in a midden, mound 5, trench 1, grid Z16, #r 14
	• Recovered from the mangrove physiographic zone.
	• Leg fragment. Height: 4.9 cm, Width: 2.9 cm, Length: 3.8 cm and walls between 6 mm y 7 mm.
	• The surface was smooth and painted.
	• Associated with the Inguapí ceramic complex and paint decoration.
	• In site was found 3 gold threads and 38 foils with thicknesses between 0.05 mm and 0.2 mm of gold, silver and, copper alloy with platinum.
**IN04** FR-A0034 1261	• Fragment collected by Bouchard’s project, found in a midden, mound 5, these fragments were found in squares X-16, Y-17, Z-16, Z-17 and A-16, Number:1261.
	• Recovered from the mangrove physiographic zone.
	• Anthropomorphic head fragment.
	• Height: 4.4 cm, Width: 3.6 cm and irregular thickness between 8 mm y 15 mm.
	• External cream colour and compact and polished surface. The interior is black and without polishing.
	• Associated with the Inguapí ceramic complex and paint decoration.
	• In site was found 3 gold threads and 38 foils with thicknesses between 0.05 mm and 0.2 mm of gold, silver and, copper alloy with platinum.
**IN05** FR-A0034 1262	• Fragment collected by Bouchard’s project, found in a midden, mound 5, these fragments were found in squares X-16, Y-17, Z-16, Z-17 and A-16, Number:1262
	• Recovered from the mangrove physiographic zone.
	• Posterior fragment of anthropomorphic head,
	• Height: 6.9 cm, Width: 6.4 cm and thickness between 7 mm y 10 mm.
	• Colour of the exterior walls uniform cream smoothed and polished regularly and painted.
	• Associated with the Inguapí ceramic complex and paint decoration.
	• In site was found 3 gold threads and 38 foils with thicknesses between 0.05 mm and 0.2 mm of gold, silver and, copper alloy with platinum.
**CO06 y CO07** R SUP 87 (dark and, bright area)	• Fragment collected by Patiño’s project, found in a midden, surface collection.
	• Recovered from the mangrove physiographic zone.
	• Ceramic face mold restored by the archaeologist and according to personal communication the bright part was found in a dry place and the dark part in a humid place. The mold has 7 [seven] perforations on the edge, possibly used as guides to prevent the positive figure obtained from moving at the time of being molded. The concave oval shape and size allows for easy ergonomic adjustment.
	• Height: 6.9 cm, Width: 6.4 cm and thickness between 7 mm y 10 mm
	• External cream colour and black interior
	• The interior surface is compact and polished, the exterior is thick and rough. The studied mold corresponds to a face, with characteristic like the object identified as T08.
	• Associated with ordinary type ceramics, various decorations, lithics and,38 gold and platinum objects
**CO08** Cocotera Tr4 20–30 cm	• Fragment collected by Patiño’s project, found in a midden, Tr4, 20 to 30 cm depth.
	• Recovered from the mangrove physiographic zone.
	• Face fragment. The coincidence between the design of the studied mold [T06 and T07] and the fragment T08 suggest
	• Height: 5.3 cm, Width: 5.5 cm, external cream colour and black interior. Slip and painted.
	• Associated with ordinary type ceramics, various decorations, lithics and,38 gold and platinum objects
**CO09** Coc. Sub. Tr4 50–60 cm.	• Fragment collected by Patiño’s project, found in a midden, Tr4, 50 to 60 cm depth.
	• Recovered from the mangrove physiographic zone.
	• Face fragment.
	• Height: 4.8 cm, Width: 5.0 cm
	• External cream colour and black interior. Slip and painted.
	• Associated with ordinary type ceramics, various decorations, lithics and,38 gold and platinum objects

ID numbers are ordered in the following sequence: Top—Identification used in this work, Bottom—Original designation.

**Fig 3:**
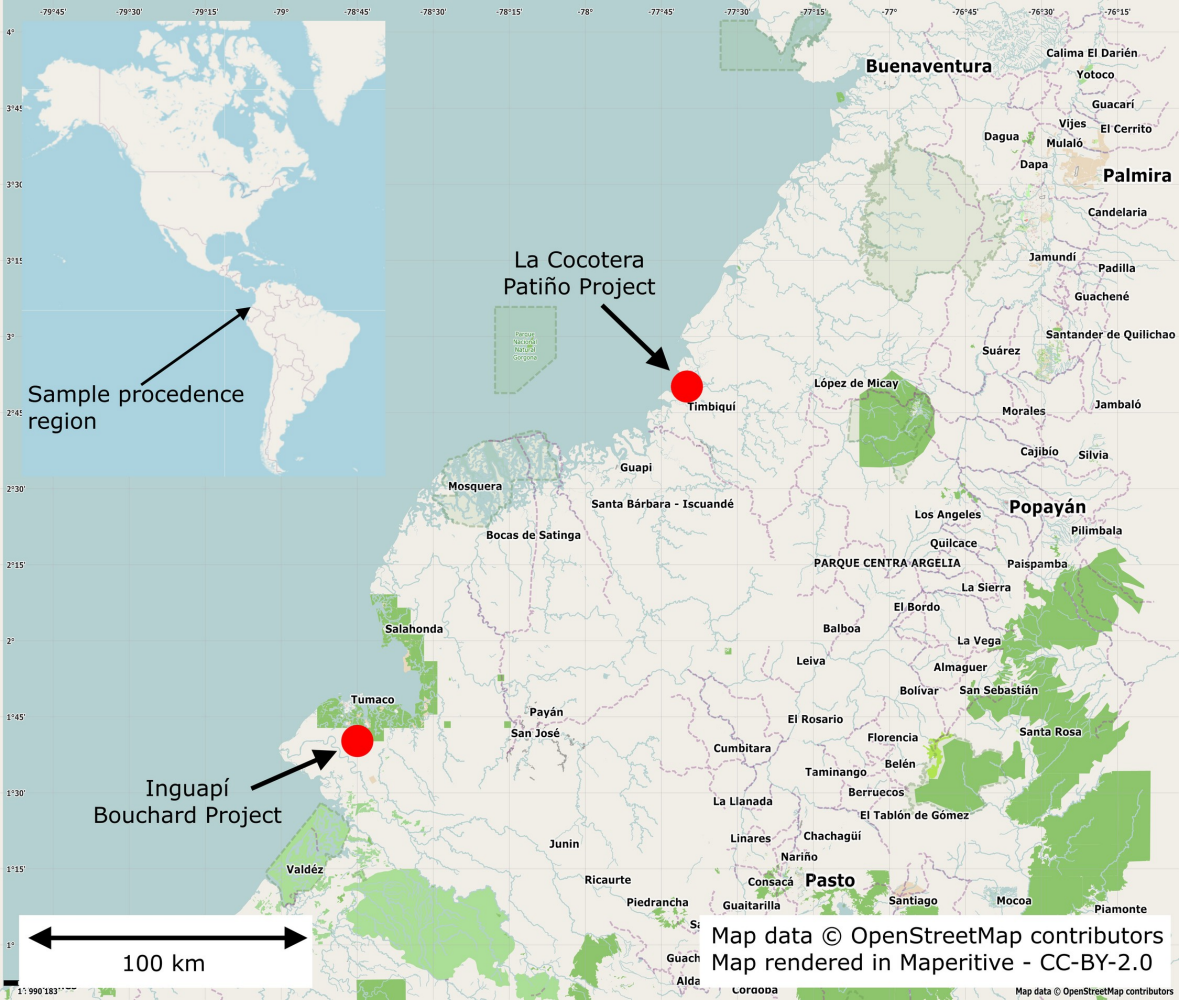
Map showing the archaeological sites where the ceramic fragments were obtained. The fragments from Inguapí correspond to IN01 to IN05. The fragments from La Cocotera correspond to CO06 to CO09. Source: Map data © OpenStreetMap contributors and rendered in Maperitive.

The selected samples come from two archaeological projects in the Tumaco region, which are of importance due to the number of excavated sites and the amount of recovered material (Fig 3). The first one is “Archaeological excavations in the Tumaco region, Nariño, Colombia” led by Jean-François Bouchard during 1977–78 (samples IN01 to IN05) [10, 25] The second group corresponds to the “Guapi-Timbiquí Archaeological Project, Pacific Coast” conducted by Diógenes Patiño between 1986–1988 (samples CO006 to CO09) [13].

The fragments IN01 to IN05 were recovered at the Inguapí site (1°40’47.7 "N 78°45’34.1” W, Latitude: 1.679914 and Longitude: -78.759483), located in the municipality of Tumaco, department of Nariño, southwest of Colombia. This site is in the Inguapí estuary of the same name and belongs to the mangrove area. The material selected for this research is part of a set of 51 figurines and 38 metal foils typical of the region, made of gold with copper and small amounts of platinum. These objects were found in strata 3 and 4 of a midden located at the base of the largest *tola*, in a set of 10 mounds with pre-Columbian occupations. Charcoal samples from these strata yielded the following dates: Ny639: 2220 ± 85BP (431–41 calBC at 94.9% probability) and Ny640: 2000 ± 80BP (203 calBC– 241 calAD at 95.4% probability) (both dates calibrated with OxCal v.4.3.2, IntCal 13) [10, 25]. The specimens belong to the ICAHN´s reference collection of the Archaeology Laboratory and they are deposited in a permanent repository, which is located in the following address: Calle 12 # 2–41, Bogotá D.C. Colombia, where they available to researchers and students (for inquiries, please refer to the website www.icahn.gov.co).

The sherds identified as CO06 to CO09 are part of the ceramic’s library of the anthropology department of the Universidad del Cauca, located in the following address: Calle 2 # 1A-25, Urbanización Caldas, Popayán, Cauca, Colombia (for inquiries, please refer to the website: www.unicauca.edu.co). The samples were collected at La Cocotera site (2°50’09.8 "N 77°41’38.9” W, Latitude: 2.83605 and Longitude: -77.694139). This site is located on the right bank of the mouth of the Bubuey River and belongs to the mangrove ecological zone. It was the most important site recorded in the survey since 49% of the total recovered material were collected here, including 110 ceramic figurines. Among the analyzed material, CO08 and CO09 fragments were found in Trench 4 TR-4, with depths between of 20–30 cm and 50–60 cm below surface, respectively. An associated charcoal sample form the site reported the date Beta20603: 1840±60 (52–335 calAD). The fragments CO06 and CO07 correspond to a mold and were recovered from the site surface. Small goldwork elements were also found in association with these ceramic figurines, with complex designs made in gold with platinum [13].

Before analyzing the specimens, all necessary permits were obtained for the described study, which complied with all relevant regulations. The corresponding numbers are: ICAHN– 132 3837 for sample No. IN01 to IN05, which was obtained on August 17th, 2016, and by the Delivery Certificate issued by The Universidad del Cauca, on November 10th, 2020 for sample No. CO06 to CO09.

During *Stage 2* (Fig 1), the methodological problem focuses on how to obtain evidence of the manufacturing process that reveals technological activities or choices, and, how to articulate and compare the evidence for each stage of manufacture. The limited information available on archaeological ceramics in Colombia, particularly from the technological perspective (non-existent for the archaeological tradition under study), limits the analysis and comparison with other technologies, regions and other traditions, despite the fact that ceramics constitute the largest material record present in the archeology of Colombia.

In *Stage 3* of (Fig 1), “structural hierarchies” were identified. Under this concept, taken from materials science and engineering, objects are deconstructed into structural units at different scales (millimeters, micrometers, nanometers, angstrom) and are integrally connected under the light of analytical, physical, mathematical or computational models or experimental evidence [39]. The identification of structural hierarchies was made using experimental measures and analytical tools, indicated in Fig 1, points 3.1 and 3.2, respectively. The experimental measurements (3.1) used depend on the scale or structural hierarchies, and the analytical tools (3.2) allowed the comparison of the data obtained from the experimental measurements performed with analytical, chemical, physical, and statistical models.

This information allowed the reconstruction of the structural hierarchies in terms of "Formulation of manufacturing hypotheses", in *Stage 4* (Fig 1). The formulation of hypothesis was based on the powder technology model, which is currently the most common method of manufacturing ceramic artefacts as well as some metals (powder metallurgy), and which includes four processing stages: 1) obtaining and preparing the raw material, 2) shaping or geometry, 3) thermal treatment and 4)finishing [40]. This research used quantitative specifications and technical parameters for each of the stages as used by powder technology, for the reconstruction of the manufacturing process, indicated in Fig 1 as the methodological stages 4.1, 4.2, 4.3 and 4.4.

The structural hierarchies that provided evidence for the *raw material hypothesis* (4.1) were the identification and quantification of the hierarchies at an atomic level performed by energy dispersive spectrometry (EDS) attached to a scanning electron microscope (SEM) and X-ray fluorescence (XRF), which determined the chemical composition of the analyzed material. X-ray diffraction (XRD) complemented the chemical composition, identifying hierarchies at a level of crystalline structures, which contributed information about the mineralogical composition of the studied material.

The identification of hierarchies at a microstructural level was performed with optical microscopy using petrographic analysis. For this purpose, thin sections were prepared and studied under plane polarized light (PPL) and crossed polarized light (XP), focusing on identification of the mineralogical phases. Descriptive or texture analyses were used to determine abundance, morphology, size fraction, spacing of inclusions and pores. All the above was performed using the petrographic methodology developed by different researchers and AnalYSIS 5 image analyzer software [41, 42].

The XRF equipment used was Rigaku brand, model ZSX Primus I, with a 4 kW Rhodium anode tube and a wavelength dispersive spectrometer. We used this equipment in standardless mode, to obtain concentrations by theoretical calculation using fundamental parameters (FP) and an internal sensitivity library with an overlap correction function. The maximum error calculated was 5%. Table 2 lists the analytical conditions used. The results are average of three measurements on areas of 10 mm diameter on unprepared surfaces of the ceramics.

**Table 2:** Analytical conditions used for XRF analysis.

Elements	Electric potential [kV]	Power [mA]	Cristal
Heavy	50	60	LiF1
Ca, K	40	75	LiF1
Cl, S, P	30	100	Ge
Mg, Na, F	30	100	RX25
Si, Al	30	100	PET

SEM-EDS analyses were performed on carbon-coated polished blocks, on which the plastic fraction or matrix and inclusions (heterogeneous areas) were characterized according to the methodology suggested and used by different researchers [43].The conditions used for the analysis are listed in Table 3 and the results are average of measurements with a 95% confidence interval.

**Table 3:** Analytical conditions used for EDS-SEM measurements.

Description	Analysis condition used
**Acceleration voltage**	20 kV
**Working distance**	10 mm
**Acquisition time**	100 seg
**Processing time**	4.8
**Measurement interval**	30 determinations
**Setting and optimization standard**	Cobalt standard
**Data verification**	Basalt standard / comparable with the analyzed material

For the XRD analyses, a Rigaku model ZSX UltimaII, with Cu anode and power 1.6 Kw was used. The specific conditions of the analyses are listed in Table 4. The XRD analyses were performed on powder samples of the original fragments and on some samples used for the refiring test. This last experimental approach considers that when the minerals that make up the clay are subjected to thermal treatments, they undergo alterations such as the change in their crystalline structure and atomic spacing as a result of progressive hydroxylation [44]. Hence, identifying the mineral phases originally present and documenting how some decompose and others form in refiring experiments at different temperatures provides information on the firing temperatures reached during the original manufacture [45].

**Table 4:** Analytical conditions used for XRD measurements.

Description	Analysis condition used
**Configuration**	Bragg-Brentano
**Voltage**	40 kV
**Power**	40 mA
**Range**	2ϴ/ ϴ:5°-100°
**Step**	0.05°/1s
**Divergence grid height**	3 mm
**Boundary grid width**	2 mm
**Anti-scatter and reception grilles position**	Open

The structural hierarchies that provided evidence for the *shaping hypotheses* (4.2, Fig 1) were macrostructural examination of the object surfaces. The analysis of the surface looked for traces of the work on the clay such as: coil or strips, joints by pinching incision channels, manual pressure to shape ergonomics, among others. The evidence was recorded with naked eye, Dino-Lite portable digital microscope and, stereoscopic magnifier Olympus SZX9 (SM).

The structural hierarchies that provided evidence for the *heat treatment hypotheses* were the crystalline phases, the microstructure, and the samples surface, particularly the color, (Fig 1, step 4.3)]. XRD mineralogical analysis was used to estimate the maximum firing temperature using as evidence the susceptibility to thermal change of some of the crystalline phases present in the raw material [46]. Likewise, at microstructural level and using SEM, vitrification was observed on fresh fractures. Uniformity or differences were observed in the surface coloration and the sample cross section. These characteristics were taken as evidence of the atmosphere, time and type of firing used.

The structural hierarchies that provided evidence for the formulation of the *finishing stage hypotheses*, by which the figurines were given the appearance observed, were the objects micro and macrostructure. At the microstructural level, smoothing patterns were observed which indicated mechanical of abrasion or polishing; at the macrostructural level, paint remains, and the use of slip were identified. The equipment used to get the evidence for this hypothesis was an Olympus SZX9 stereoscopic magnifier (SM) with a DP12 digital camera.

## Results, analysis, and formulation of manufacturing hypotheses

The analytical results will be presented providing technical details for each of the manufacturing stages defined by the powder technology, in accordance with Fig 1, number 4.

### Raw material hypothesis

The first stage of the ceramic manufacturing process comprises the chemistry, mineralogy, raw material selection and its preparation, mixing and homogenization process. Therefore, the results obtained provide technical details that constitute evidence of these sub-processes and respond to activities or technical decisions.

#### Chemical analysis by XRF

Table 5 lists the results obtained by XRF, which were grouped by provenance and correspond to the chemical composition determined in the samples expressed as oxides.

**Table 5:** Results of the bulk chemical composition obtained by XRF, presented as stoichiometric percentages by weight.

Compound	Inguapí Fragments					La Cocotera Fragments			
	IN01	IN02	IN03	IN04	IN05	CO06	CO07	CO08	CO09
**Na**_**2**_**O**	nd	0.5	0.9	1.0	1.0	nd	nd	nd	nd
**MgO**	nd	0.7	0.7	0.8	0.8	1.0	0.7	0.3	0.5
**Al**_**2**_**O**_**3**_	23.1	26.2	23.5	21.3	23.9	16.6	14.7	19.2	17.6
**SiO**_**2**_	60.9	54.9	60.2	58.4	56.6	52.3	47.0	45.8	48.7
**P**_**2**_**O**_**5**_	0.2	0.3	1.1	0.5	3.6	1.5	1.1	1.6	3.4
**SO**_**3**_	nd	0.1	0.1	nd	nd	0.4	0.5	2.7	2.2
**Cl**	nd	nd	nd	nd	nd	0.3	0.4	0.4	0.4
**K**_**2**_**O**	0.6	0.6	0.7	0.7	0.7	2.1	1.8	2.2	2.6
**CaO**	2.5	2.7	3.3	1.7	3.2	5	4.2	5.9	6.4
**TiO**_**2**_	1.3	1.7	1.2	0.8	1.2	3.1	3.3	5.7	4.3
**MnO**	0.3	0.1	0.02	1.0	0.2	0.4	2.5	0.2	0.2
**Fe**_**2**_**O**_**3**_	10.8	11.9	8.1	13.7	8.7	17.1	23.5	15.5	13.6

Conventions = nd: element analyzed and not detected. All samples were detected below 0.1% Sr and Zr.

**Fig 4:**
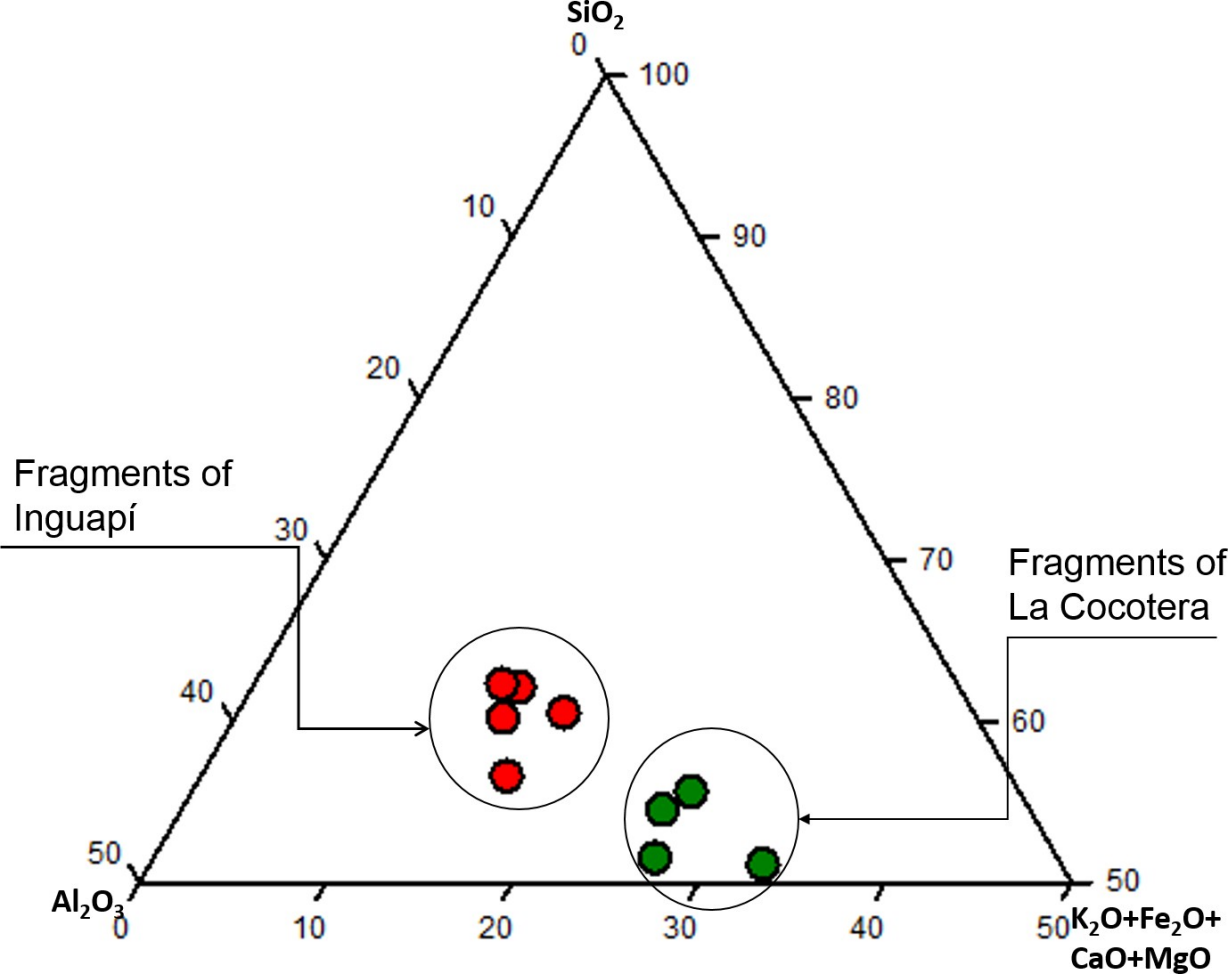
Ternary composition diagram of the analyzed samples. The bulk concentrations of alumina, silica and the sum of CaO + MgO + K_2_O and Fe_2_O_3_ were plotted.

Table 5 shows the following differences between sites: first, the bulk silica and alumina levels for La Cocotera fragments are lower (46–52% and 15–19%, respectively) than those for Inguapí (55–60% and 21–26%). Conversely, La Cocotera samples report higher contents of alkali and alkaline earth metal oxides (Na_2_O, K_2_O, MgO and CaO). Iron oxide levels are high in both sites, but especially so at La Cocotera (21–28% Fe_2_O_3_, compared to 13–17% at Inguapí), where they are also matched by remarkably high titania (average 4.1% compared to 1.2% at Inguapí). These differences are evidenced in the ternary composition diagram shown in Fig 4.

#### Mineralogical analysis by XRD

**Fig 5:**
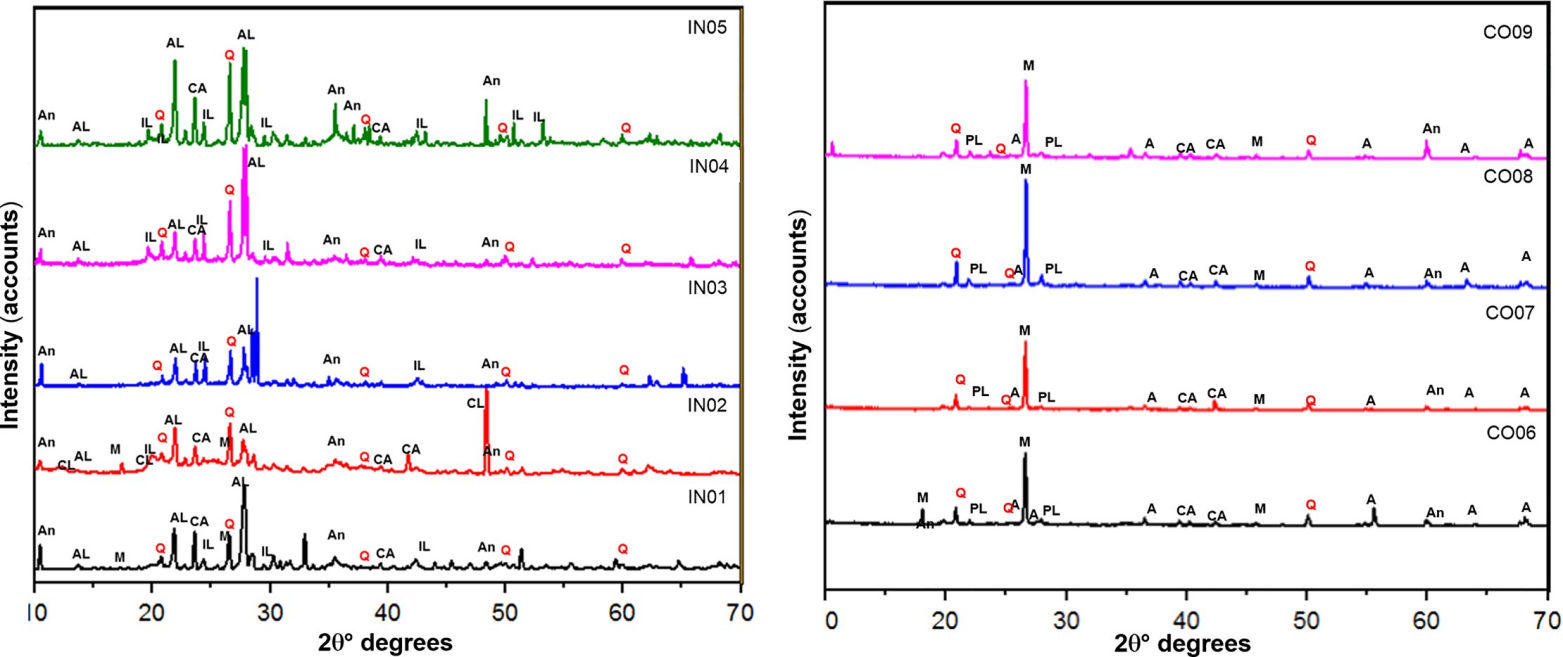
Diffraction patterns obtained from the samples. A. corresponds to fragments IN01, IN02, IN03, IN04 and IN05 and B. corresponds to fragments CO06, CO07, CO08 and CO09. Conventions indicate: An (amphibole: hornblende), PL (plagioclase), M (mica: muscovite), Q (quartz), IL (illite), CL (chlorite), CA (kaolinite), A (anatase), Mt (montmorillonite) and AL (albite).

The results obtained by XRD showed the presence of kaolinite Al_2_(Si_2_O_5_)(OH) in all samples, but clearly allowed the discrimination between two different mineralogical groups: in the first group, samples from Inguapí presented illite and kaolinite clay minerals, with sodium feldspar (albite)], amphiboles (hornblende) and quartz (Fig 5A). In the second group, which comprised La Cocotera ceramic samples, montmorillonite and kaolinite clay minerals were detected, along with plagioclase, amphiboles (hornblende), mica (muscovite), quartz and anatase (Fig 5B). Interestingly, the few sherds with anatase were showed a white (lighter) coloration [47, 48]. Overall, it can be concluded that the detected elemental and mineralogical differences indicate the use of two different types of raw material sources for the elaboration of the same representation of ceramic objects.

#### Microstructural analysis by light microscopy

**Fig 6:**
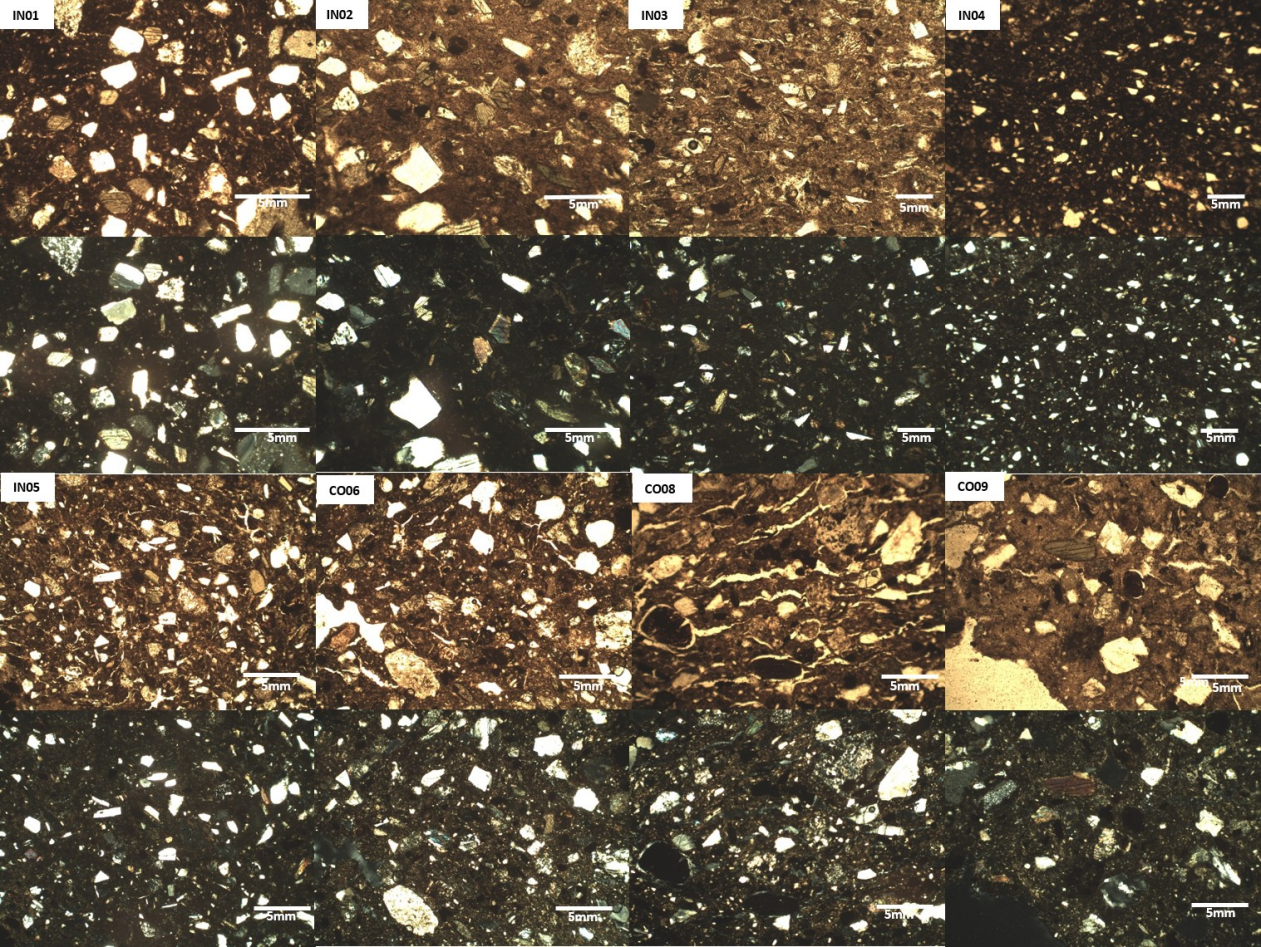
Microphotographs of samples petrographic thin sections. Inguapí samples[(IN) under PPL and XP and La Cocotera samples (CO) under PPL and XP.

Fig 6 show the petrographic thin sections of samples PPL and XP, in which compact and homogeneous microstructures were not observed, the above indicated the use of unrefined materials. Regarding the inclusion, an abundance between 20% and 30% of non-plastic inclusion was estimated, among which we could identified quartz, feldspar, mica, amphibole, rock fragments, pumice, volcanic glass, some opaque minerals in the thin sections. Based on the frequency and angular shape or geometry of quartz, we can suggest intentional addition and previous crushing in thin sections Their addition improves refractory properties and reduces volumetric change in clays due to the thermal effect [49, 50].

The main differences in the thin sections were the size of inclusions, pores shape and type of some inclusions. In Inguapí (IN) potsherds, the fine inclusions fraction (determined between 20 and 140 microns) represented an average of 90% of the section, while in sherds from La Cocotera [CO], the fine fraction was less than 60%, Table 6 lists the quantitative characterization of aplastic inclusions. The difference in size is an indicator of a probable selection of material that was used as temper for the figurines made in Inguapí, namely fine quartz rich sands a choice not made at La Cocotera. Regarding pore shape the pores in the IN group where fine-sized and presented vesicular shapes (Fig 6, IN), while for the CO group these were planar and/or vough shaped and showed a greater length (Fig 6, CO). It may be proposed that the pores left after firing in Inguapí samples correspond to organic matter naturally present in the raw material, while those from La Cocotera could correspond to the microstructural change caused by the crystalline transformation of quartz (SiO_2_)when fired to temperatures above ~575°C. The third difference corresponds to the presence of clay pellets for La Cocotera group of samples, (Fig 6, CO), which were not detected in the Inguapí group (Fig 6, IN). The presence of this material could be the result of a lack of mixing or incomplete kneading of the ceramic paste.

**Table 6:** Quantitative characterization aplastic inclusions.

M.	Frequency[%]	Tipe	Range [μm]	Fine fraction			Coarse fraction		
				Size [μm]	[%]	Mode	Size [μm]	[%]	Mode
T01	30	Unimodal	20–550	20–210	84	50–70	220–550	16	---
T02	20	Unimodal	20–360	20–200	91	80–100	210–360	9	---
T03	30	Unimodal	20–140	20–100	91	50–60	110–140	9	---
T04	30	Unimodal	20–300	30–170	97	50–60	180–300	3	--
T05	30	Unimodal	20–300	20–170	90	60–70	180–300	10	--
T06	20	Bimodal	20–560	20–140	47	60–80	150–560	53	180–200
T08	30	Bimodal	20–560	20–140	68	60–70	150–560	32	180–200
T09	30	Bimodal	20–650	30–180	38	120–150	190–650	62	270–300

#### Chemical and microstructural analysis by Scanning Electron Microscopy with Energy Dispersive Spectrometer (SEM-EDS)

**Fig 7:**
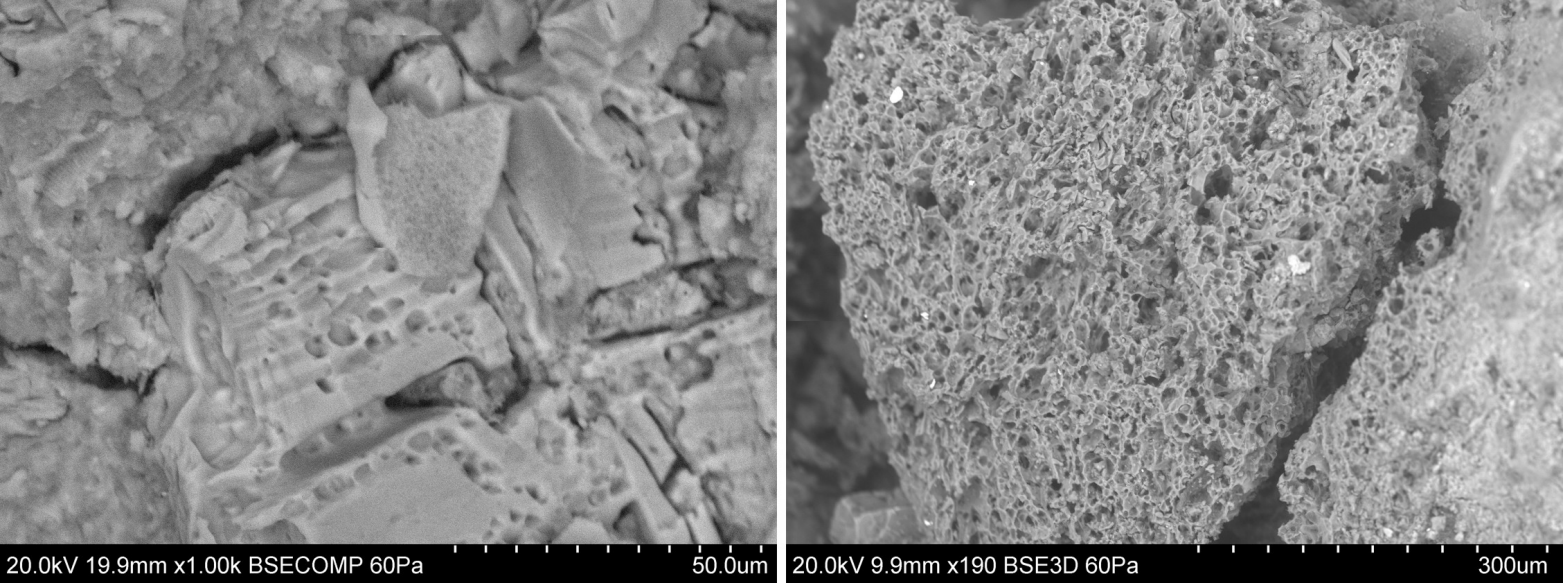
Volcanic inclusions in the fresh fracture of IN04. A. Volcanic glass. B. Pumice found on the fresh face of fragment IN04.

Volcanic glasses and pumice were morphologically identified in the polished samples and fresh fractures of all the samples. Fig 7A shows, fragments of volcanic glass and 7B is pumice, both figures belong to the IN04 sample.

The average of the chemical composition of 5 volcanic glasses inclusions of each sample by SEM-EDS is shown in Table 7 95% confidence interval. Their presence and chemical composition are consistent with the volcanism of the Andean zone between Colombia and Ecuador [51].

**Table 7:** Results of the chemical composition obtained by SEM-EDS of volcanic glasses presented as stoichiometric percentages by weight compound.

	Inguapí Fragments															La Cocotera Fragments								
	IN01			IN02			IN03			IN04			IN05			CO06			CO08			CO09		
**Na**_**2**_**O**	4.1	±	0.5	4.5	±	0.8	4.8	±	0.3	3.7	±	0.6	3.7	±	0.3	7.8	±	0.2	10.3	±	1.1	3.8	±	0.2
**MgO**	0.3	±	0.1	0.5	±	0.2	0.6	±	0.2	1.2	±	0.6	0.5	±	0.1	0.9	±	0.1	1.5	±	0.2	0.2	±	0.0
**Al**_**2**_**O**_**3**_	13.4	±	1.0	14.3	±	2.1	15.2	±	1.2	13.5	±	1.6	16.7	±	1.0	16.7	±	0.7	20.4	±	2.1	12.5	±	0.7
**SiO**_**2**_	76.1	±	1.6	74.2	±	2.9	71.9	±	2.3	74.0	±	2.2	71.1	±	1.6	73.3	±	2.3	63.3	±	3.3	77.3	±	1.0
**P**_**2**_**O**_**5**_	0.9	±	0.0	2.0	±	0.0	nd			1.4	±	0.4	2.1	±	0.1	nd			nd			nd		
**K**_**2**_**O**	1.9	±	0.7	1.3	±	0.5	2.0	±	0.2	2.3	±	0.5	1.5	±	0.1	0.2	±	0.0	1.2	±	0.1	3.6	±	0.2
**CaO**	2.0	±	0.6	2.0	±	0.6	3.0	±	0.5	1.2	±	0.4	2.0	±	0.4	nd			1.4	±	0.7	1.3	±	0.4
**TiO**_**2**_	0.4	±	0.1	0.3	±	0.0	0.6	±	0.1	0.4	±	0.1	0.3	±	0.0	nd			0.5	±	0.3	0.3	±	0.1
**FeO**	1.1	±	0.4	0.9	±	0.3	1.9	±	1.1	0.3	±	0.7	2.1	±	0.1	1.0	±	0.1	1.3	±	0.2	1.2	±	0.1
**CrO**	nd			nd			nd			2.0	±	0.0	nd			nd			nd			nd		

Conventions = nd: element analyzed and not detected.

**Fig 8:**
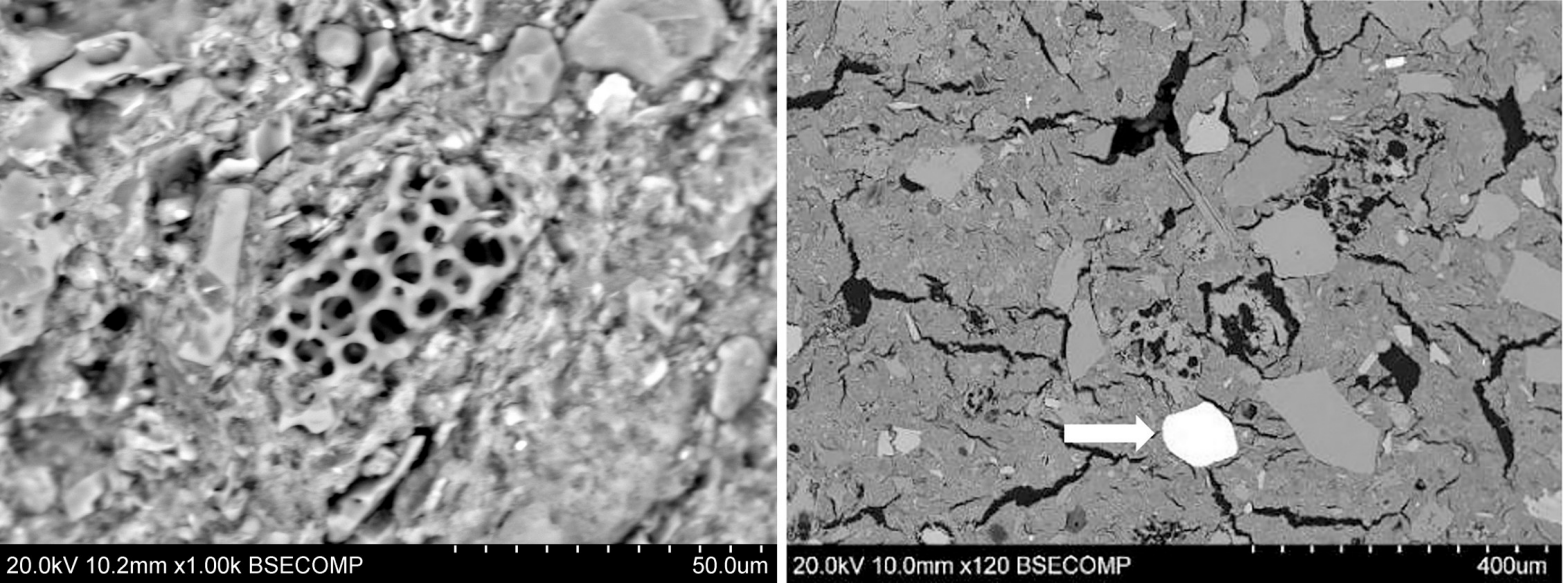
Inclusions detected in the samples. A. Diatom present in IN04 and B. Iron sulfide detected CO08.

Additionally, diatoms were detected in the Inguapí fragments, of the type and morphology shown in Fig 8A. Although the presence of these inclusions of organic origin is not uncommon in archaeological ceramics, their detailed and interdisciplinary study has provided archaeological information of great interest related to provenance, sources, and manufacturing technology [52]. Of note here is their presence exclusively in IN samples. Conversely, iron sulfide inclusions (Fig 8B) were identified only in CO samples

In summary, the results obtained allow us to formulate a hypothesis for the first manufacturing step, namely "raw material". It is proposed that the two groups of figurines, corresponding to the two archaeological sites, were made with two types of raw material, as indicated by their chemical, mineralogical and microstructural differences. Possibly, there were also two strategies types of raw material selection, based on the differences in the percentage of fine material found, and in the preparation of the ceramic paste, as suggested by the presence of clay pellets in only one of the groups. These differences are reflective of the origins and initial processing of the raw material and thus reflect technological choices related to the availability of raw materials and the recipes followed by the potters.

### Shaping hypothesis

The second stage of the manufacturing process is the shaping, i.e. the method used to give shape or geometry to the figurines. Since this process engages the plastic properties of the ceramic paste macrostructural scale analyses are the basis for the hypothesis formulation. Archaeological evidence of the shaping of ceramic figurines using two techniques (molding and modeling or the combination of these) can be observed in the early phases of this tradition [7, 9, 25]. By molding we understand the shaping technique that identical objects are reproduced by pressing the clay paste on a mold and, by modeling is worked with hands a clay pellet until achieving the desired shape.

#### Macroscopic analysis

**Fig 9:**
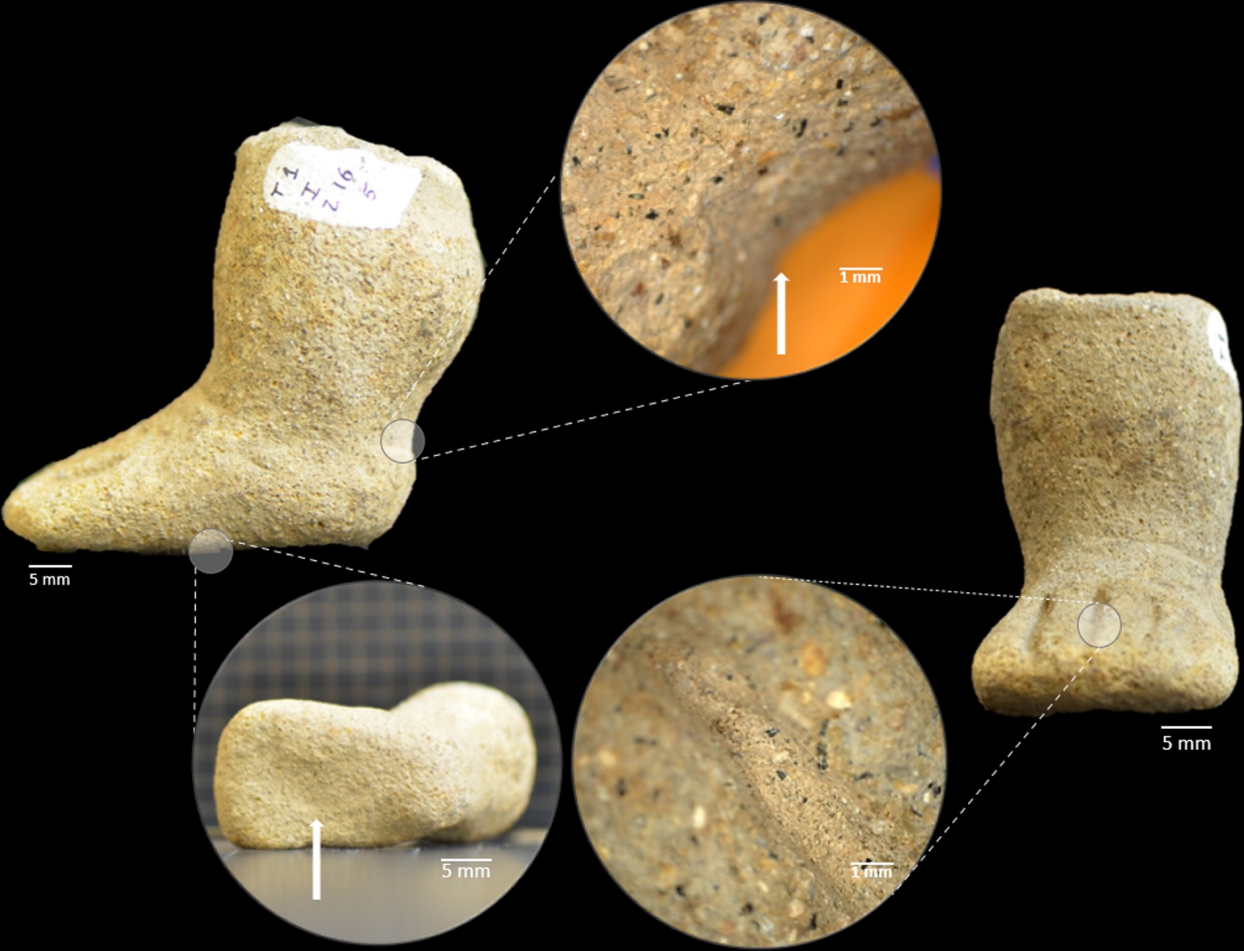
Evidence of direct modeling on clay. IN01 fragment showing traces of direct modeling on the clay, manual pressure of the fingers to shape the ankle, pointed out with white arrows and, sole of the foot and incised decoration to represent the fingers.

Evidence of direct modeling on clay to manually shape the ankle and sole of the foot (Fig 9, white arrows), was found in sample IN01, taken as representative of this group of figurines. In addition, applied decoration is observed in the bracelet in IN02 and the eyes in IN04 (see Fig 2). Finally, incisions were used to demarcate features such as fingers (IN01), legs (IN03) and ears (IN05). In these figurines, the incision is clean, without a ridge or excess material, which indicates that this operation was performed on the clay in a semi-dry state.

The evidence observed in fragments CO08 and CO09, such as the concave geometry and traces of finger impressions left on the back of the objects, suggest that the technique used was molding. Moreover, the detailed optical analysis of the mold identified as CO06 and CO07, and of matching features in figurine fragment CO08, suggest that this object was indeed shaped using the mold studied.

As an addition to this, we suggest that the potters from Inguapí and La Cocotera made technological decisions related to the ceramic paste making and the forming technique. The analyzed samples from Inguapí were shaped manually by modelling, for this purpose homogeneous ceramic pastes were made with selected fine temper, kneading and mixing it properly. On the contrary, a lower manual contact required for modeling caused the ceramic paste of the figurines of La Cocotera to be thicker, coarser and poorly blended. In summary, the results obtained using the proposed methodology allowed the following hypothesis to be formulated for the shaping stage: two different shaping methods were used to give shape or geometry to the figurines. Evidence from macrostructural (direct modeling traces to shape the clay and applique decoration) examination indicates that the figurines from Inguapí were shaped by modeling. Conversely, macrostructural evidence (concave shape and pressure marks on the back of the reproductions) from the figurines from La Cocotera indicate that they were shaped by molding.

### Heat treatment hypothesis

The identification of the structural hierarchies of this stage of the process contributed to the knowledge of the firing parameters. Particularly, information was obtained on the pyrotechnology and the technological choices involved in this stage of the process.

#### Analysis of heat-induced transformation of crystalline phases by XRD

Table 8 presents a summary of the firing temperature estimates, including the crystal phases identified by XRF, the estimated temperature according to the transformed phase, and bibliographic references to the experimental supporting such estimations.

**Table 8:** Results by XRD and maximum estimated firing temperature.

ID	Mineral phases detected in the original samples	Maximum Estimated firing temperature [C°]	Transformed phase/Reference
**IN01**	Illite, Kaolinite, Albite, Muscovite, Quartz and Hornblende	IN01 < 586	Quartz [53]
**IN02**	Illite, Chlorite, Kaolinite, Albite, Muscovite, Quartz and hornblende	IN02 < 650	Chlorite [54, 55]
**IN03**	Illite, kaolinite, albite, quartz, and hornblende.	700°C <IN03<800°C	Quartz [53]
**IN04**	Illite, kaolinite, albite, quartz, and hornblende.	IN04 < 800°C	Illite [54]
**IN05**	Illite, kaolinite, albite, quartz, and hornblende.	IN05 < 800°C	Illite [54, 56]
**CO06**	Montmorillonite, kaolinite, plagioclase, muscovite, quartz, and anatase	CO06 y CO07 < 586°C	Quartz [53]
**CO07**	Montmorillonite, kaolinite, plagioclase, muscovite, quartz, and anatase	CO06 y CO07 < 600°C	Quartz [53]
**CO08**	Montmorillonite, kaolinite, plagioclase, muscovite, quartz, and anatase	CO08 < 586°C	Quartz [53]
		CO08 < 600°C	
**CO09**	Montmorillonite, kaolinite, plagioclase, muscovite, quartz, and anatase	08 < 600°C	Quartz [53]

**Fig 10:**
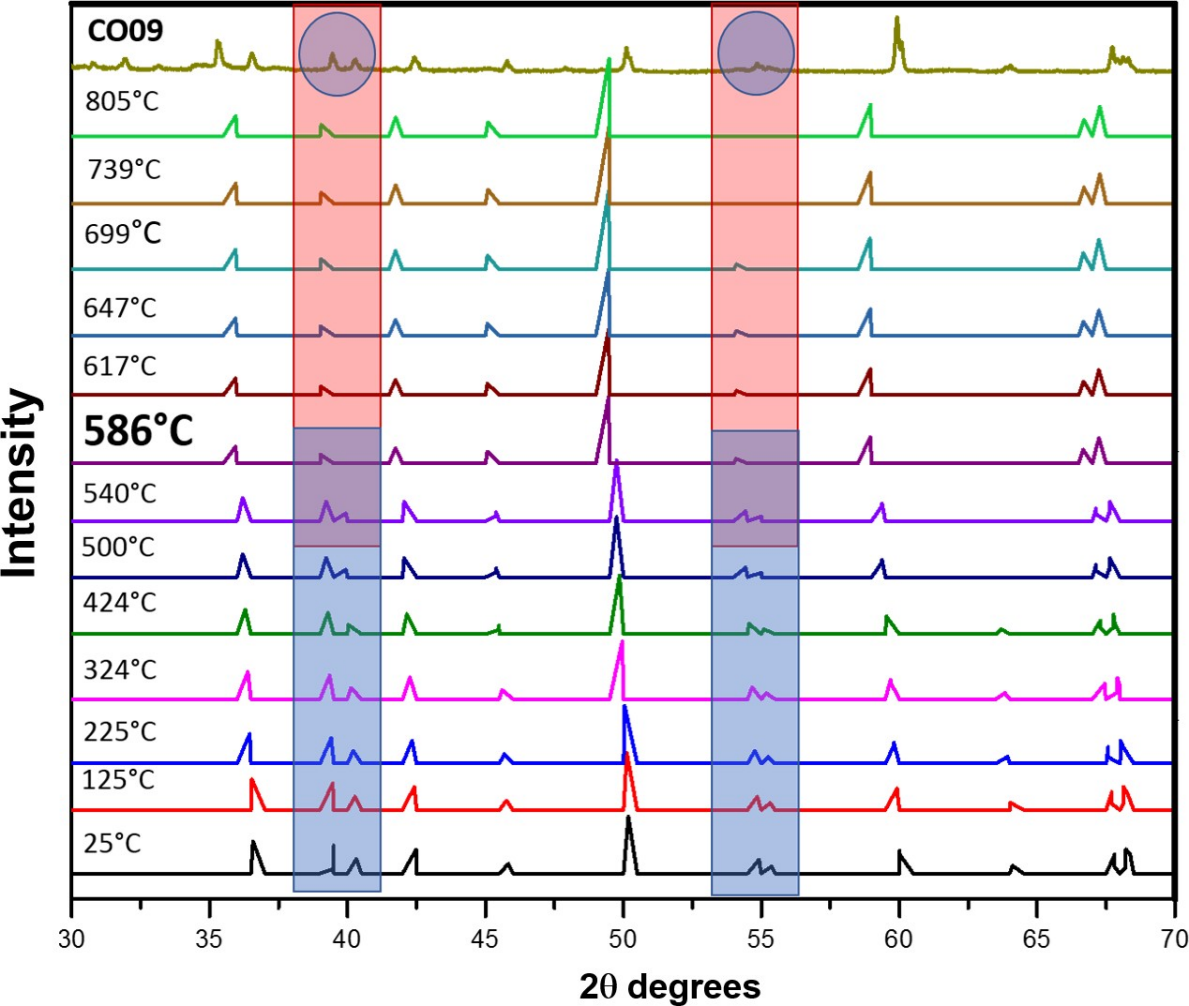
Comparison of CO09 diffraction patterns with quartz at different temperatures [43]. The similarities in the colored areas revealed in the overlap of colors (rectangular and circular) allow us to estimate the firing temperature of the CO09 figurine between 540°C and 586°C.

Our firing temperature estimates were based on the crystalline transformation by thermal effect of quartz, chlorite and illite. The crystalline transformation of SiO_2_ quartz from the α-ß phase at 572.9°C [846K] was used as a reference for estimating the temperature of samples from figurines IN01, CO06, CO07, CO08 and CO09, all estimated to be less than 600°C. Fig 10 illustrates the reasoning process by comparing the diffraction pattern of sample CO09 sample to those of quartz experimentally heated from 24°C to 805°C [53]. The areas of the diffractogram where the quartz inversion is reflected are highlighted.

**Fig 11:**
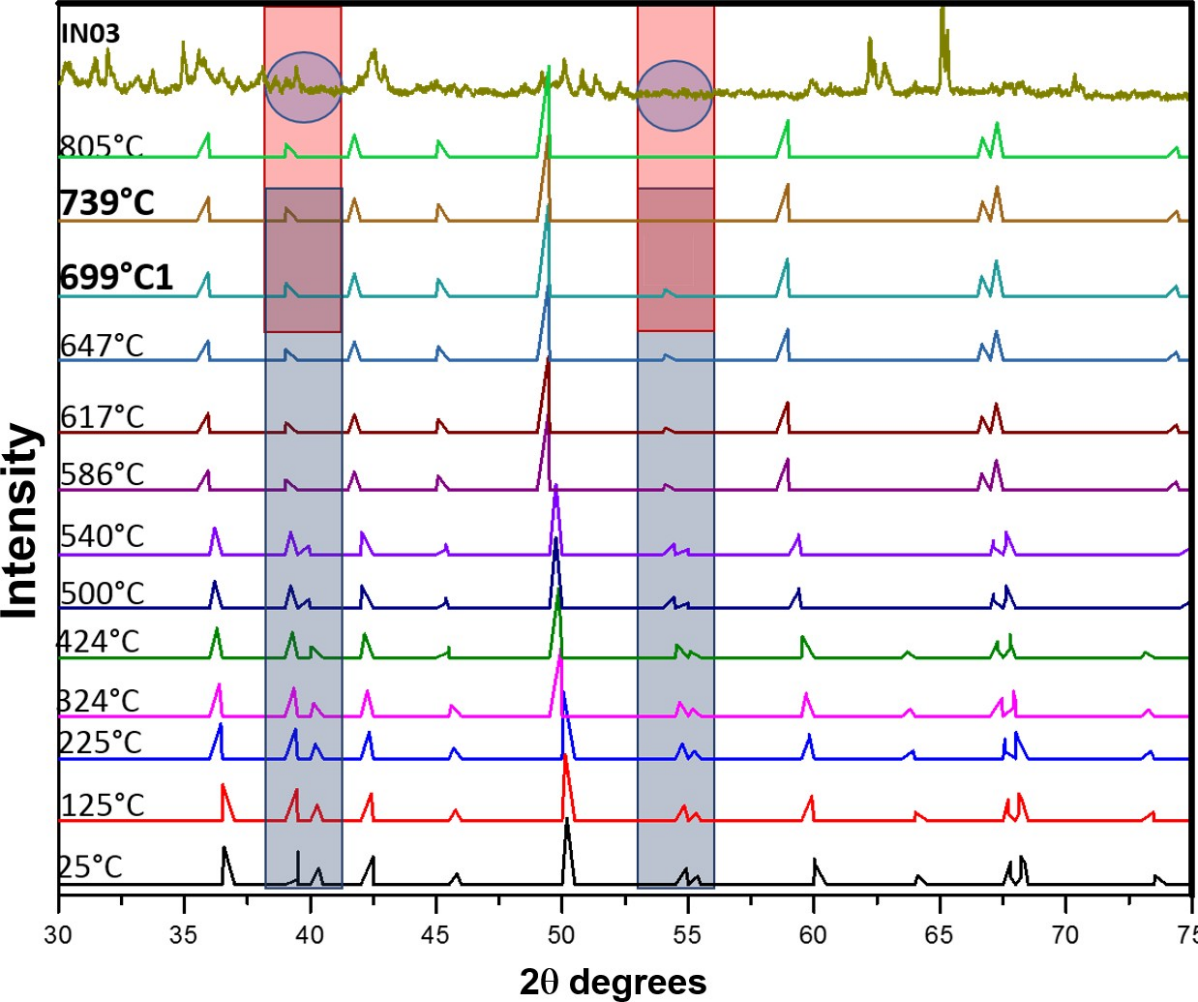
Comparison of the IN03 diffraction patterns with quartz at different temperatures [43]. The similarities in the colored areas revealed in the overlap of colors (rectangular and circular) allow us to estimate the firing temperature of the IN03 figurine between 699°C and 739°C.

Fig 11 shows a similar approach to sample IN03. The similarities can be appreciated in the color overlap zones as a result of a diffraction signal decrease [13] by 55.32°, starting at 699°C and disappearing at 739°C, which is taken as the estimated temperature range for this sample.

The second transformation was appreciated in IN02 and was taken from the archaeometric and experimental works performed at archaeological sites [54, 55]. These works reported that under oxidizing conditions, chlorite begins its thermal transformation by decomposition at 500°C, while reflection [1] disappears at 650°C. The diffractogram for sample IN02 shows this reflection, which indicates that ceramic was not subjected to temperatures above 650°C.

The last transformation was detected in the diffraction patterns of samples IN04 and IN05 and particularly in the presence of reflection at 4.48 Ǻ (19.77 2θ) that corresponds to illite. According to experimental works, this reflection suffers a gradual decrease in intensity until it disappears at 1000°C [56]. Maritan *et al*. 2006] showed that this decomposition occurs at much lower temperatures, close to 800°C [54]. From this we derive that our samples did not exceed 800°C.

#### Macro- and microstructural analysis by LM and SEM

**Fig 12:**
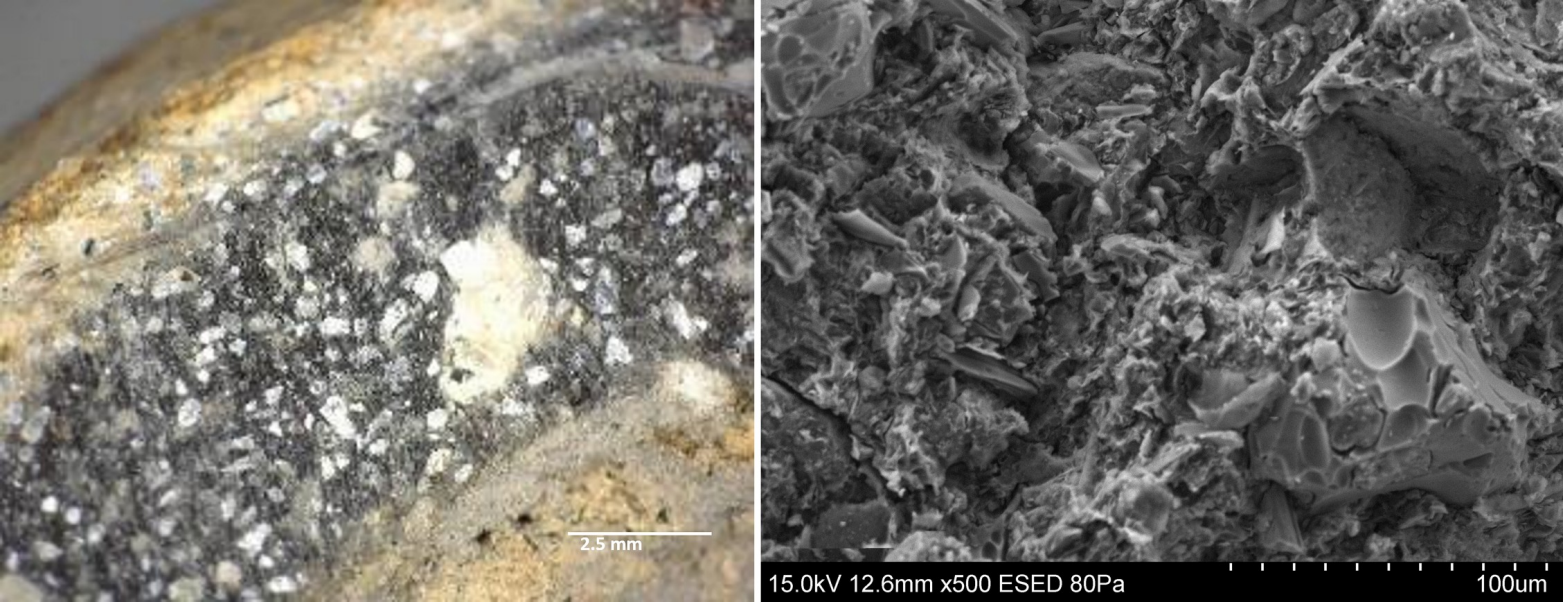
Micro and macrostructural evidence of heat treatment in sample IN01. A. Cross section showing the complete section with bright edges and a dark core. B. SEM image showing the microstructure without vitrification.

Fig 12A shows a cross-section of IN01, displaying a dark core coloration with bright exterior surfaces; Fig 12B shows the microstructure this same sherd under higher magnification, where individual clay minerals and the lack of vitrification can be noticed. These color differences reveal a thermal gradient, the use of an oxidizing atmosphere, and an incomplete oxidation process of organic matter and iron. This is characteristic of open fires where the maximum temperature is low and/or held for short periods only [45, 57] These characteristics were common to all the figurines analyzed.

Finally, a dark patch was observed on the external face of the fragment in figurine CO09, resembling a slight smoky trace that is suggestive of direct exposure to a flame, as also typical of an open fire or bonfire firing system.

In summary, the results obtained using the proposed methodology allowed to reconstruct the "heat treatment" stage and reveal technological choices resulting in different maximum firing temperatures. The IN figurines were fired at temperatures below 800°C, while the CO fragments estimated temperatures did not exceed the 600°C. However, the microstructural and macrostructural evidences suggest all the ceramic objects here examined were fired in an open fire structures, in an oxidizing atmosphere and at relatively low temperatures.

#### Finishing hypothesis

**Fig 13:**
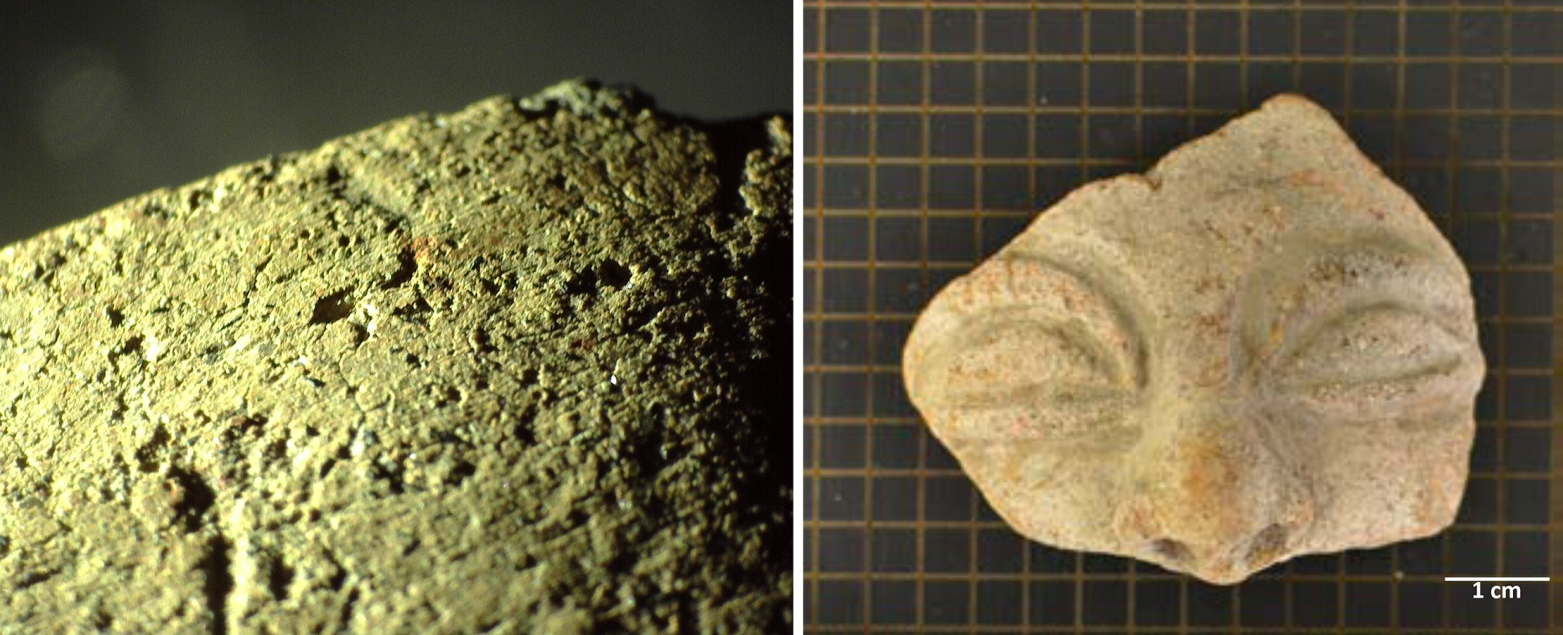
Evidence of finishing on the analyzed fragments. A. Traces of fine and regular polishing observed in IN05 used to smooth the surface of the piece. B Presence of slip in CO08.

Evidence of the finishing stage is manifest in the final appearance of the object, though it should be noted that in some cases it could be carried out before firing. Relevant evidence was obtained at the macrostructural level. Among the evidence common to IN and CO, we point out the presence of traces of red paint–a frequent feature in ceramic figurines of this tradition. Among the differences, we note the traces of parallel lines, suggestive of smoothing or superficial polishing, which are only present in IN samples (Fig 13A). Although samples CO8 and CO09 show a smooth and regular surface, such linear patterns were not observed. Instead, in CO samples we documented the presence of traces of a milky white slip (Fig 13B)

## Conclusions

**Fig 14:**
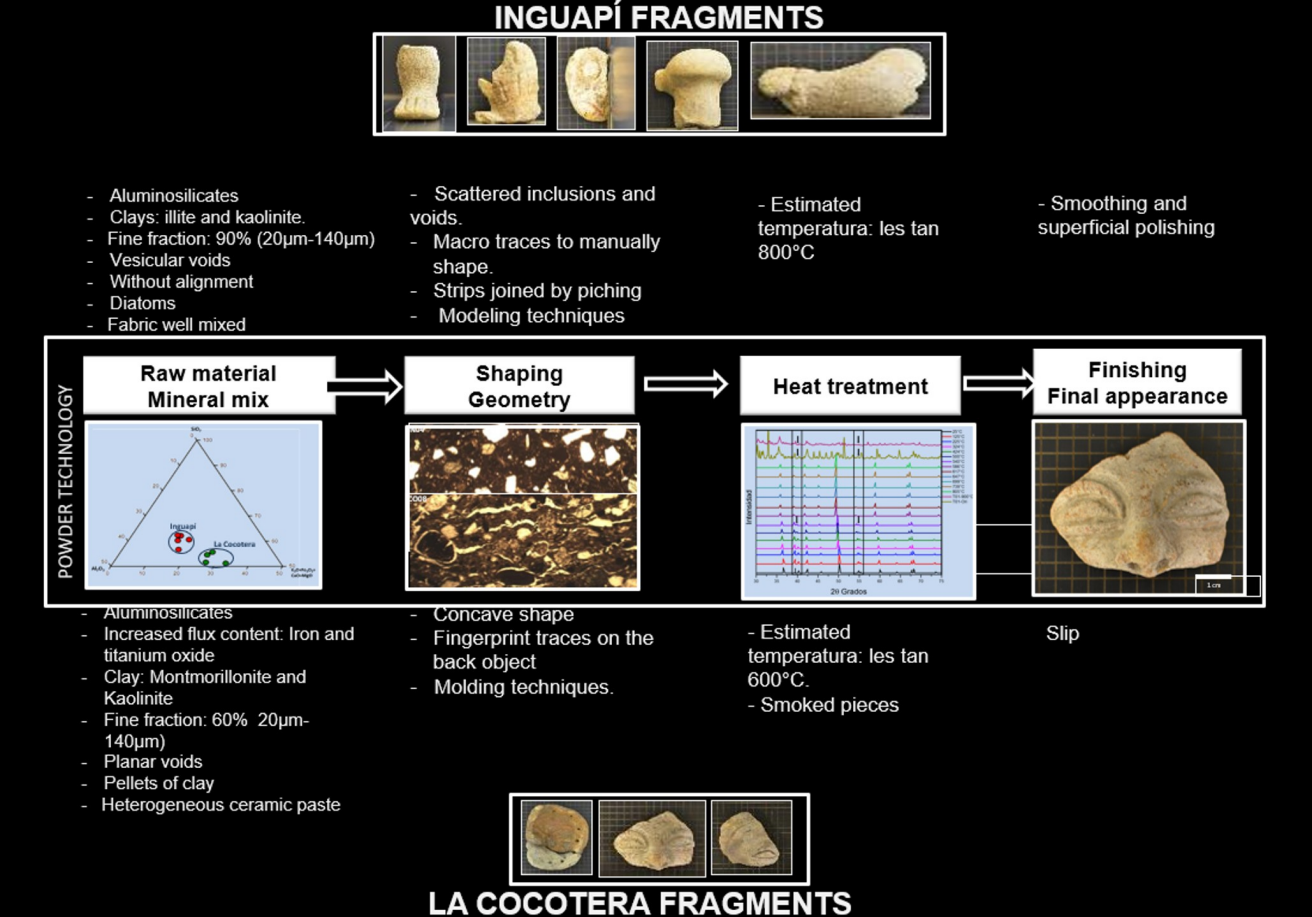
Differences in the manufacturing stages of ceramic figurines from the Tumaco archaeological tradition.

The use of reverse engineering as a methodological tool allowed the materials characterization of the Tumaco ceramic figurine fragments, and the formulation of hypotheses regarding their manufacture. Our results identified clear and consistent differences in the manufacturing processes leading to the same type of representation (ceramic figurines), which indicate distinct technological choices at each of the two sites analysed. The differences were detected in each and every one of the manufacturing stages, as summarized in Fig 14, thus representing two different manufacturing pathways or *chaînes opératoires* within the broader Tumaco ceramic figurine tradition.

The consistent differences in the manufacturing process of these ceramic figurines during the most technologically complex phase of the Tumaco tradition are indicative of technological choices by the manufacturers, and the development of local ceramic traditions. It is likely that these figurines were produced at workshops at, or near, the sites where they are recovered, adapting to local raw materials, customs and knowledge. Despite sharing a style that reflects a shared ideology, technological choices reflective of a standardized industry are not observed. Further studies should aim to validate our hypotheses while expanding the range of sites to compare ceramic technologies within and among the different archaeological traditions of Colombia and Ecuador, including other ceramic types and exploring possible cross-craft interactions with metallurgy and other technologies. This will develop our understanding of ancient technologies and their interaction, while providing a complementary perspective on the development of social complexity and central political structures in the broader region.
